# Association hyperthyroïdie et cancers différenciés de la thyroïde

**DOI:** 10.11604/pamj.2016.24.18.7605

**Published:** 2016-05-06

**Authors:** Nassim Essabah Haraj, Hayat Ahandar, Siham El Aziz, Asma Chadli

**Affiliations:** 1 Service d'Endocrinologie et de Maladies Métaboliques, CHU Ibn Rochd, Faculté de médecine et de pharmacie, Université Hassan II Casablanca, Maroc

**Keywords:** Hyperthyroïdie, cancer, basedow, nodule toxique

## Abstract

La présence d'une hyperthyroïdie n'est plus une assurance contre la survenue d'un cancer thyroïdien. L'association des deux n'est pas rare. Il s'agit d'une étude rétrospective de 355 dossiers de patients suivis pour cancer différenciés de la thyroïde au service d'endocrinologie du CHU Ibn Rochd entre 1986 et 2014, on retrouve douze patients suivis pour hyperthyroïdie chez qui une association fortuite avec un cancer différenciés de la thyroïde a été découverte sur l'examen anatomopathologique, soit une prévalence de 3.38%. L’âge moyen à la découverte est de 44,8 ans, avec une nette prédominance féminine (8/12). Huit patients avaient un nodule toxique, 3 goitres basedowifiés et un cas de maladie de basedow. Tous ont bénéficié d'une thyroïdectomie totale. Il s'agissait de carcinome papillaire chez tous les patients. Le microcarcinome était le plus prédominant (6 patients). Un carcinome insulaire était retrouvé chez une patiente, avec présence de métastases rachidiennes et rétro orbitaire. Un traitement par l'iode radioactif a été indiqué chez cinq patients. Le diagnostic de l'hyperthyroïdie n’élimine pas la possibilité d'un cancer thyroïdien associé. La malignité doit toujours être gardée à l'esprit et de ce fait proposer une démarche diagnostique comparable à celle établie pour tout nodule thyroïdien.

## Abstract (en)

The presence of hyperthyroidism is no longer an insurance against the occurrence of thyroid cancer. The combination of the two is common. This is a retrospective study of 355 files of patients followed for differentiated thyroid cancer in the endocrinology department at CHU IBN ROCHD from 1986 to 2014. 12 of those patients were followed for hyperthyroidism, and a fortuitous association with differentiated thyroid cancer was found during the anatomopathological exam, giving us a 3.38% prevalence. The average age of discovery is 44.8 years, with a marked female predominance (8/12). Eight patients had a toxic nodule, 3 had Basedow's goiters, and one had Graves’ disease. All underwent total thyroidectomy. In all patients, the cancer was a papillary carcinoma. Microcarcinoma was the most predominant (6 patients). An insular carcinoma was found in a patient with spinal and retro-orbital metastases. Treatment with radioactive iodine was prescribed to five patients. The diagnosis of hyperthyroidism does not eliminate the possibility of an associated thyroid cancer. Malignancy should always be kept in mind and therefore lead to a diagnostic approach comparable to that for any thyroid nodule.

## Introduction

L'association d'une hyperthyroïdie à un cancer de la thyroïde est une association non rare. La prévalence est variable dans les études (entre 2.3 et 13.6) [1]. Cette association reste controversée. Est-ce que la thyrotoxicose protège contre le cancer? Ou est ce qu'il s'agit d'un facteur de risque de cancer? Ou alors d'une association fortuite? Nous rapportons les observations de patients suivis pour hyperthyroïdie, et chez qui le diagnostic de cancer a été découvert de façon fortuite. A travers ces observations, les différents aspects cliniques et thérapeutiques de cette association seront étudiés.

## Méthodes

Nous avons mené une étude rétrospective des dossiers de patients suivis pour carcinome différencié de la thyroïde au service d'endocrinologie du CHU IBN Rochd entre 1986 et 2014. Durant cette période, 355 patients avec cancer différencié de la thyroïde ont été suivis. Parmi eux, 12 présentaient une hyperthyroïdie en préchirurgie. Le diagnostique de cancer thyroïdien a été découvert de façon fortuite chez 11 patients et suite à l'apparition de métastases rachidienne et rétro-orbitaire chez une patiente. Les différentes variables étudiées: les caractéristiques cliniques, histologiques et thérapeutiques.

## Résultats

**Figure 1:**
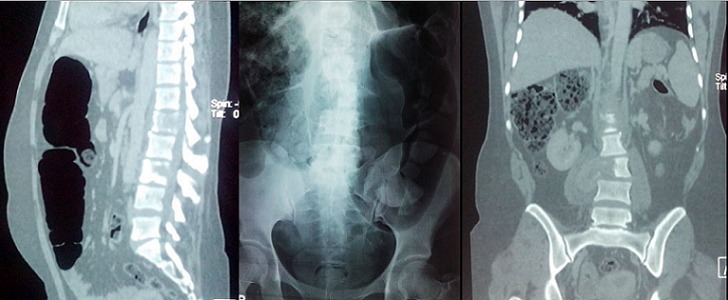
Lyse osseuse en rapport avec des métastases rachidiennes chez la patiente 10

**Figure 2:**
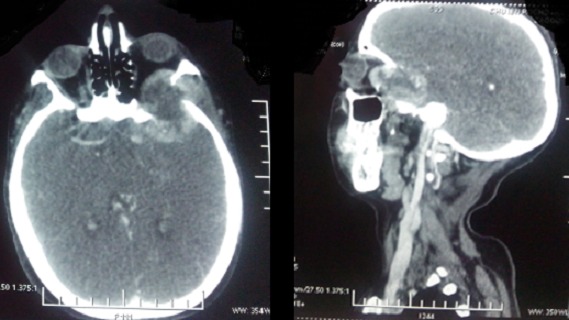
TDM cérébrale de la patiente 10 objectivant les métastases cérébrales rétro-orbitaires

Parmi les 12 patients, 8 avaient un nodule toxique, 3 un goitre basedowifié, et 1 cas de maladie de basedow. L’âge moyen des patients était de 44,8 ans. Prédominance féminine 4H/8F. Le Tableau 1 résume les caractéristiques des patients ayant une association d'hyperthyroidie et cancer différencié de la thyroïde. Chez 11/12 de nos patients le carcinome était de bon pronostic et l’évolution n'a pas montré de récidive avec une durée de suivi de 1 à 8 ans. Le type histologique prédominant était le microcarcinome papillaire. Un traitement par l'iode radioactif a été indiqué chez 5 patients et réalisé chez 4 patients. L’évolution était défavorable chez une patiente (patiente 10) qui avait un carcinome papillaire insulaire, découvert après apparition de localisations secondaires (métastase rachidienne et rétro-orbitaire) (Figure 1, Figure 2). Après la thyroïdectomie totale, les sites métastatiques étaient inopérables chez elle, et un traitement par chimiothérapie au niveau des sites métastatiques suivie d'un traitement par l'iode radioactif était préconisé, mais l’évolution a été marquée par le décès après 4 mois sans avoir fait d'IRAthérapie.

**Tableau 1:** Caractéristiques des patients avec association hyperthyroïdie et cancer différencié de la thyroïde

Patients	Age/sexe	Chirurgie	Etiologie d'hyperthyroidie	Diagnostic histologique
1	55/F	Thyroïdectomie totale	Nodule toxique	Carcinome papillaire intra-nodulaire
2	35/F	Thyroïdectomie totale	Nodule toxique/GMHN	4 microcarcinomes papillaires (lobe droit, isthme, lobe gauche). En dehors du nodule toxique
3	48/F	Thyroïdectomie totale	Nodule toxique/GMHN	Nodule encapsulé. Carcinome papillaire de 1,5 cm
4	50/F	Thyroïdectomie totale	Nodule toxique/GMHN	Microcarcinome papillaire de 6mm extra nodulaire
5	63/F	Thyroïdectomie totale	Goitre basedowifié	Microcarcinomes papillaire de 4 mm
6	53/F	Thyroïdectomie totale	Goitre basedowifié	Microcarcinome papillaire de 5 mm
7	48/H	Thyroïdectomie totale	Goitre basedowifié	4 microcarcinomes papillaires (2 lobes droits + 2 lobes gauches)
8	24/F	Thyroïdectomie totale	Nodule toxique/GMHN	Microcarcinome papillaire de 3mm de grand axe
9	48/H	Thyroïdectomie totale	Maladie de basedow	Microcarcinome papillaire
10	39/F	Thyroïdectomie totale	Nodule toxique	Carcinome papillaire avec composante insulaire non encapsulé avec emboles et effraction de la capsule.
11	58/H	Thyroïdectomie totale	GMHN toxique	2 microcarcinomes de 5mm
12	17/H	Thyroïdectomie totale	Nodule toxique	carcinome papillaire de lobe droit intranodulaire de 4cm encapsulé sans images d'emboles

## Discussion

L'association d'hyperthyroïdie et cancer thyroïdiens est une association non rare. La prévalence est variable dans les études. La fréquence des cancers de la thyroïde retrouvée chez des patients suivis pour hyperthyroïdie et traités chirurgicalement est variable, allant jusqu′à 21,1% [1]. Cela est probablement dû à plusieurs facteurs: la cause de l′hyperthyroïdie, les différents critères permettant de choisir la chirurgie comme traitement radical, l′étendue de la thyroïdectomie (lobectomie ou une thyroïdectomie totale), et la fréquence des microcarcinomes découverts fortuitement. Tous les types histologiques de cancers de la thyroïde peuvent être associés à l′hyperthyroïdie. Le plus fréquent est le carcinome papillaire de la thyroïde. L'association avec le carcinome anaplasique et médullaire est exceptionnelle [2, 3], cela est dû aussi à la faible incidence de ces cancers en général.

L’étiopathogénie de l'association carcinome thyroïdien et hyperthyroidie reste controversée. De nombreux travaux ont été consacrés aux relations entre le cancer thyroïdien et l'hyperthyroïdie. Trois circonstances ont été décrites [4]: **Hyperthyroïdie juxtanéoplasique:** où l'hyperthyroïdie est le fait du tissu thyroïdien sain adjacent au cancer. C'est la situation la plus fréquente. Le cancer est en règle hypo fonctionnel.**Hyperthyroïdie néoplasique:** où l'hyperthyroïdie est liée à l'activité fonctionnelle du carcinome thyroïdien primitif ou de ses métastases. Il s'agit de nodules toxiques malins dont une vingtaine de cas ont été rapportés dans la littérature. Leur diagnostic est évoqué souvent a posteriori quand l'hyperthyroïdie persiste ou récidive après thyroïdectomie totale. Ceci peut correspondre au cas de la patiente 10 dans notre série, mais l'aggravation importante de son état générale et l’évolution rapide vers le décès n'a pas permis de compléter les investigations pour confirmer le diagnostic. **Hyperthyroïdie paranéoplasique:** où l'hyperthyroïdie peut être liée à la sécrétion d'une substance TSH-like par une tumeur extrathyroïdienne comme les tumeurs trophoblastiques. Cette substance est capable de se fixer sur le récepteur de la TSH et induire une thyrotoxicose par stimulation du tissu thyroïdien normal [4].

Concernant la maladie de basedow, il existe une différence significative dans l'incidence du cancer de la thyroïde. Chez les patients avec maladie de basedow variant entre 5 et 15%. Certaines études suggèrent l'existence d'une association, vu que l'incidence chez les patients avec maladie de basedow est plus élevée que dans une population en euthyroïdie. [1]. En ce qui concerne les nodules chauds, des mutations activatrices du récepteur de la TSH ont été identifiées dans certaines études, pouvant suggérer l'existence d'une association non fortuite [1, 5, 6]. En effet, la pathogénie de l'association hyperthyroïdie cancer de la thyroïde reste mal élucidée et soulève un bon nombre de questions d'ordre physiopathologique. [4]. Concernant la classification histologique. La plupart des cancers associés à une hyperthyroïdie sont petits [7, 8]. Il s'agit dans 88% des cas de cancers détectés dans la maladie de Basedow de cancers = 10 mm [7–9], le suivi de ces patients a montré qu'ils ont un excellent pronostic [10]. D'autres études sont contradictoires et suggèrent le caractère agressif du cancer chez ces patients suivis pour basedow. De grande cohorte prospective peuvent donner des réponses plus claires à ces questions [1]. Dans notre étude, on retrouve que chez les patients avec association hyperthyroïdie et carcinome différencié de la thyroïde il s'agit majoritairement de microcarcinome et qu'ils ont généralement un bon pronostic.

## Conclusion

Notre série confirme que la probabilité de découvrir un cancer chez les hyperthyroïdiens n'est pas rare. La malignité doit toujours être gardée à l'esprit et de ce fait proposer une démarche diagnostique comparable à celle établie pour tout nodule thyroïdien. Il y a une absence d'orientation clinique et para-clinique pré-thérapeutique. Et Il s'agit dans la majorité des cas de cancer de bon pronostic.

### Etat des connaissances actuelle sur le sujet

- La probabilité d'un cancer de la thyroïde dans un contexte d'hyperthyroïdie est faible.

### Contribution de notre étude à la connaissance

- L'association hyperthyroïdie et cancer de la thyroïde n'est pas rare;
- Il s'agit souvent d'une association fortuite.
